# Effects of Pirfenidone on Peripheral Nerve Healing in an Experimental Sciatic Nerve Injury Model in Rats

**DOI:** 10.7759/cureus.116853

**Published:** 2026-09-24

**Authors:** Murat Ö Yay, Zahir Kizilay, Nesibe Kahraman Çetin

**Affiliations:** 1 Neurological Surgery, Faculty of Medicine, Aydın Adnan Menderes University, Aydın, TUR; 2 Pathology, Faculty of Medicine, Aydın Adnan Menderes University, Aydın, TUR

**Keywords:** nerve regeneration, nf-kappa b, peripheral nerve injuries, pirfenidone, sciatic nerve

## Abstract

Introduction: Pirfenidone is a molecule that has recently been tested in extrapulmonary diseases owing to its notable anti-inflammatory and antioxidative mechanisms of action, in addition to its antifibrotic characteristics. The objective of this investigation was to elucidate the impact of pirfenidone administration on the restorative processes in a rat model subjected to peripheral nerve injury.

Methods: In the present investigation, 32 rats were randomly allocated into four groups (n=8 per group). A standardized crush injury was induced in the right sciatic nerve by applying an aneurysm clip for five minutes in two groups, one of which received pirfenidone. A 10-mm sciatic nerve segment encompassing the injury site was harvested for histopathological evaluation. On days 15 and 30, the sciatic functional index (SFI) was calculated. Histopathological evaluation included injury grading, axon count, and immunohistochemical staining for Caspase-3 and nuclear factor kappa B (NF-κB).

Results: On day 15, no statistically significant difference was observed between the Injury + Pirfenidone and Injury groups in SFI values (p=0.086). However, by day 30, SFI values were significantly higher in the Injury + Pirfenidone group than in the Injury group (p<0.001). Caspase-3 and NF-κB immunoreactivity was numerically lower in the Injury + Pirfenidone group, but neither difference reached statistical significance (p=0.195 and p=0.161, respectively).

Conclusion: Pirfenidone treatment was associated with improved functional recovery at day 30 following experimental sciatic nerve injury. Although Caspase-3 and NF-κB immunoreactivity was numerically lower in the pirfenidone-treated group, these differences were not statistically significant and therefore do not establish an anti-apoptotic or anti-inflammatory mechanism. Further studies are needed to clarify the mechanisms underlying the observed functional improvement.

## Introduction

Peripheral nerves are more prone to injury due to their long courses. Although trauma is the most common cause of injury, other factors such as infections, ischemic conditions, ionizing radiation, external compression, difficult childbirth, inflammation, and electrical shocks can also contribute to nerve damage [1]. After peripheral nerve trauma, inadequate healing, abnormal regeneration of the nerve, and the consequent loss of functional capacity in the muscle group that the nerve frequently stimulates, as well as chronic pain, are common consequences, regardless of the injury model [2]. Therefore, the primary goal in post-injury treatment is to minimize functional loss and restore nerve integrity and functionality as effectively as possible [3].

Numerous experimental studies have been conducted to accelerate the healing process and bring the nerve back to its pre-injury state in the treatment of peripheral nerve injuries [4-6]. Despite countless studies, no treatment method that can restore the injured peripheral nerve to its pre-damage functional state has yet been identified. In experimental peripheral nerve injury models, evaluation of recovery requires not only morphological observation but also functional and molecular assessment. The sciatic functional index (SFI) is one of the most widely used quantitative methods for assessing motor functional recovery in rodent sciatic nerve injury models, because it reflects the integrated consequences of axonal regeneration, target muscle reinnervation, and locomotor coordination. Therefore, the SFI provides a relevant functional outcome measure for determining whether a therapeutic intervention translates into meaningful motor improvement [7,8].

Pirfenidone, also referred to as 5-methyl-1-phenyl-2(1H)-pyridone, is an orally administered agent with established antifibrotic activity and reported antioxidant and anti-inflammatory properties, including modulation of oxidative stress and reduction of pro-inflammatory cytokines such as tumor necrosis factor-alpha (TNF-α) [9]. These properties have prompted its investigation in several experimental models of tissue injury. However, evidence regarding its effects on peripheral nerve regeneration remains limited, and, to our knowledge, pirfenidone has not previously been directly evaluated in an experimental peripheral nerve injury model. Therefore, whether pirfenidone can improve functional recovery following peripheral nerve injury remains unclear.

Peripheral nerve regeneration is a complex process involving inflammatory responses, Schwann cell activity, axonal regeneration, and remodeling of the injured nerve microenvironment. Caspase-3 is a key executioner of apoptosis and has been used as a marker of apoptotic cell death in peripheral nerve injury and Schwann cell-related experimental settings [10-12]. NF-κB is a central regulator of inflammatory signaling and has been implicated in Schwann cell responses following peripheral nerve injury [13-16]. Accordingly, Caspase-3 and NF-κB were selected as complementary markers to explore apoptotic and inflammatory responses associated with peripheral nerve injury and regeneration, rather than as direct evidence of a specific mechanism of pirfenidone. Based on this rationale, the present study aimed to evaluate the effect of pirfenidone on recovery after experimental sciatic nerve injury using functional assessment by the SFI together with histopathological evaluation, axon counting, and immunohistochemical assessment of Caspase-3 and NF-κB.

## Materials and methods

Ethical statement

Subsequent to securing the requisite authorization from the Aydın Adnan Menderes University Animal Ethics Committee (approval number: 64583101/2019/140), all experimental procedures were meticulously carried out in strict adherence to the protocols outlined in the National Institutes of Health (NIH) Guide for the Care and Use of Laboratory Animals.

Animals

A total of 32 mature female Wistar albino rats, aged 12 between 14 weeks and weighing 250 ± 25 g, were included in the study. The animals were obtained from the Aydın Adnan Menderes University Experimental Animal Breeding and Research Center. They were housed under controlled environmental conditions at 20-24°C and 50 ± 10% relative humidity with a 12-hour light/dark cycle. Food and water were provided ad libitum throughout the study.

Experimental groups

The rats were randomly allocated to four experimental groups (n=8 per group) in a 1:1:1:1 ratio using a computer-generated randomization sequence:

Group 1: Control (C) - The right sciatic nerve was meticulously exposed and subsequently sutured without further intervention. (Acute nerve injury - , pirfenidone -)

Group 2: Control + Pirfenidone (CP) - The right sciatic nerve was exposed and sutured, followed by the administration of pirfenidone at a daily dose of 50 mg/kg, continuing throughout the duration of the experimental phase. (Acute nerve injury - , pirfenidone +)

Group 3: Injury (I) - The right sciatic nerve was exposed, and a localized compressive injury was induced using an FE-572K Yasargil aneurysm clip (Aesculap AG & Co., Tuttlingen, Germany) for a duration of five minutes. After the clip was removed, the injury site was sutured using 4-0 silk sutures, and the anatomical layers were reapproximated in accordance with their original structure. (Acute nerve injury + , pirfenidone -)

Group 4: Injury + Pirfenidone (IP) - The right sciatic nerve was exposed, subjected to compressive injury, and sutured, with the anatomical layers reclosed as per their native configuration. Subsequently, pirfenidone at a dose of 50 mg/kg was administered via gastric lavage once daily, continuing until the conclusion of the experimental phase. (Acute nerve injury + , pirfenidone +) The selected dose (50 mg/kg/day) was based on prior experimental studies in rats demonstrating biological activity and acceptable tolerability in various injury and fibrosis models, rather than on dose-finding studies specific to peripheral nerve injury [8,9]. For clarity, the Control (C) and Control + Pirfenidone (CP) groups underwent identical surgical exposure of the sciatic nerve without application of crush injury, whereas the Injury (I) and Injury + Pirfenidone (IP) groups were subjected to a standardized crush injury using an aneurysm clip applied for five minutes.

No animals were excluded or lost during the experimental period, and all 32 animals completed the study and were included in the final analyses.

Anesthesia and surgical procedures

After the rats included in the study were weighed, anesthesia was calculated according to their body weight and administered intraperitoneally as a combination of 50 mg/kg ketamine and 5 mg/kg xylazine. Following the administration of requisite analgesia to the rats, the surgical site was carefully depilated, and the skin was thoroughly sterilized using a povidone-iodine solution. In the ventral decubitus position, a 3 cm-long incision parallel to the right thigh was made with a scalpel. After the skin incision, the subcutaneous tissue was passed, and the muscle layers were exposed. Through blunt dissection between the femoral and gluteal muscles, the right sciatic nerve was revealed from its origin to the point where it gives off its first branch. In the Injury (I) and Injury + Pirfenidone (IP) groups, the sciatic nerve was compressed experimentally using an aneurysm clip (FE-752K aneurysm clip, Aesculap AG & Co., Tuttlingen, Germany) for five minutes, thereby creating a standardized compression and ischemic injury. This crush injury model and the five-minute duration were selected in accordance with previously established experimental protocols in the literature to ensure reproducibility and consistency of nerve damage. In the Control (C) and Control + Pirfenidone (CP) groups, the sciatic nerve was exposed in the same manner, but no crush injury was applied [17,18].

The musculature surrounding the injured region was delineated using silk sutures. Following the induced trauma, the tissue layers were reapposed in precise accordance with their native anatomical configuration. After suturing the skin, the skin was disinfected again with povidone-iodine, and the animal was allowed to wake up. The sequential surgical procedure, including preparation of the incision site (Figure 1A), skin incision (Figure 1B), exposure of the right sciatic nerve (Figure 1C), induction of sciatic nerve injury using an aneurysm clip (Figure 1D), marking of the injury site (Figure 1E), and skin closure (Figure 1F), is illustrated in Figure 1. Starting from the day of injury, 50 mg/kg pirfenidone was delivered daily to rats in the CP and IP groups via gastric lavage. Pirfenidone administration was initiated immediately after injury in order to target the early inflammatory and apoptotic phase of nerve injury. On the 30th day of the study, the rats were euthanized through cervical dislocation following the administration of appropriate analgesia under profound anesthesia.

**Figure 1:**
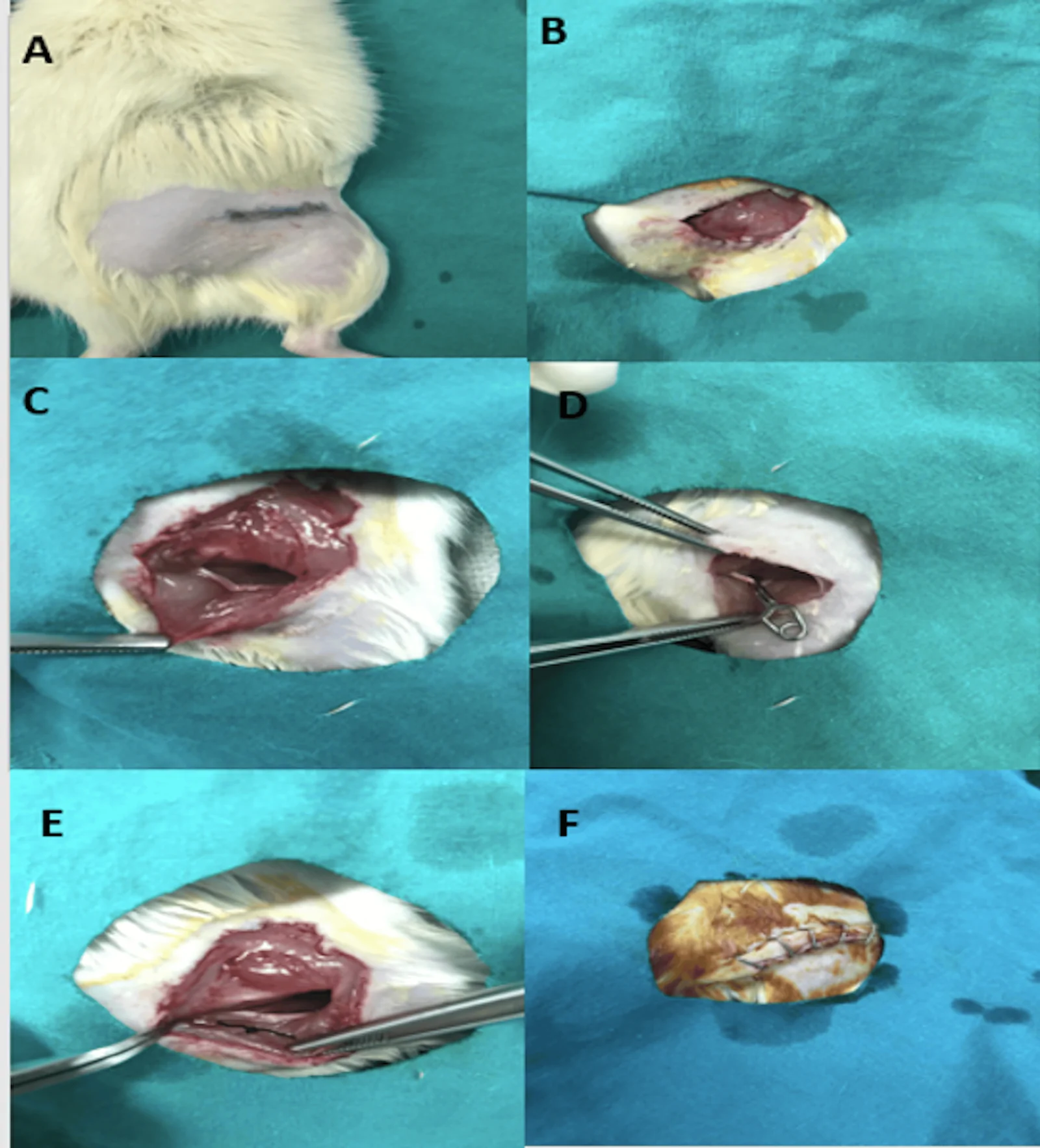
Sequential representation of the procedures applied to the rats Shaving and marking the incision site before surgery (A), after skin incision (B), exposure of the right sciatic nerve through dissection (C), induction of acute damage to the right sciatic nerve (compressed for five minutes with FE-752K aneurysm clip) (D), marking of the injury site (E), and skin suturing after surgery (F). FE-752K aneurysm clip: Aesculap AG & Co., Tuttlingen, Germany

Walking track and analysis

On days 15 and 30 of the experiment, the animals’ hind paws were coated with red food coloring and walking-track analysis was performed to assess motor function. To ensure that the rats walked in the same direction, a walking track apparatus measuring 8.2 cm in width and 42 cm in length, with 12-cm-high side walls and ending in a dark compartment, was used (Figure 2A). A representative trial of a rat traversing the walking track is shown in Figure 2B. White A4 paper sheets were placed on the floor of the walking track. Prior to testing, the rats were allowed to traverse the apparatus multiple times to familiarize themselves with the path. Subsequently, both hind paws were coated with food coloring, and the animals were encouraged to walk along the track, leaving footprints on the paper. Animals that did not produce technically adequate footprints were allowed to repeat the procedure. Measurements were obtained using an AEK-Tech digital millimeter ruler. The intermediate toe spread (ITS), toe spread (TS), and print length (PL) measurements used for SFI calculation are illustrated in Figures 3A, 3B, 3C, respectively.

**Figure 2:**
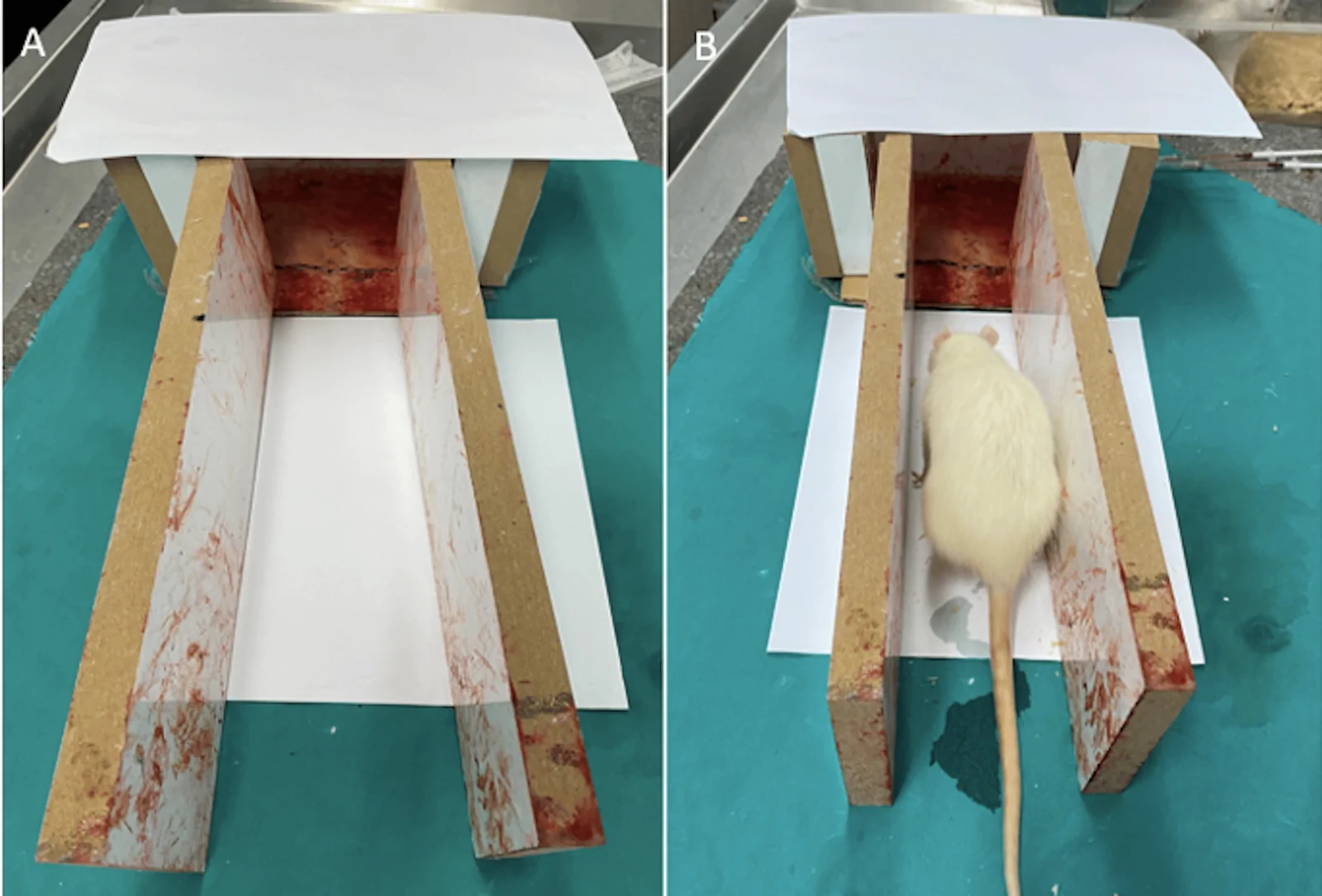
Walking track apparatus (A) and rat's trial on the walking track (B)

**Figure 3:**
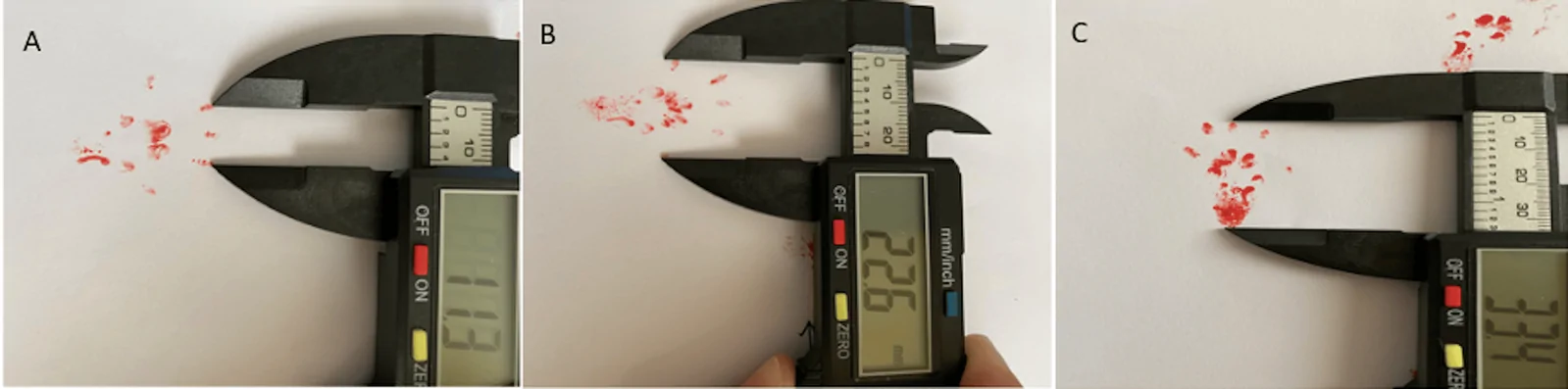
(A) Middle toe distance, (B) toe spread distance, and (C) heel-to-third toe distance

The SFI was calculated using the formula described by Bain et al. [19]. For each animal, three clearly identifiable and non-overlapping footprints were selected for analysis. Eligible footprints were required to show complete visualization of the heel and relevant toes, permitting reliable measurement of PL, TS, and ITS. Smudged, incomplete, overlapping, or otherwise technically inadequate footprints were excluded. If fewer than three eligible footprints were obtained, the walking-track procedure was repeated until three technically adequate footprints were available. For each selected footprint, three parameters were measured: the distance from the heel to the third toe (PL), the distance from the first to the fifth toe (TS), and the distance between the second and fourth toes (ITS). SFI was calculated separately for each of the three selected footprints, and the mean of the three values was used as the representative SFI value for each animal at each time point. All footprint selection, measurements, and SFI calculations were performed by an investigator blinded to group allocation. The SFI was calculated using the following equation:

*SFI=-38.3[(EPL-NPL)/NPL] + 109.5[(ETS-NTS)/NTS] +13.3 [(EIT-NIT)/NIT] - 8.8*

Where E denotes the experimental side and N represents the normal side. A value of 0 signifies intact function, whereas a value of -100 reflects complete dysfunction. The mean SFI value was derived from the measurements of these three selected footprints.

Histopathological evaluation

A 10-mm segment of the right sciatic nerve encompassing the injury site was harvested and immersed in 10% buffered formalin for pathological analysis. After fixation, the tissues were processed routinely using an automatic tissue processing system for 14 hours. The specimens were embedded in paraffin, and sections of 3-4 μm thickness were cut using a microtome (Thermo Shandon HM 430 Sliding Microtome, Thermo Fisher Scientific Inc., MA, USA).

The obtained sections were stained with hematoxylin-eosin (H-E), Toluidine blue, and Masson’s Trichrome for routine histopathological evaluation and assessment of axonal degeneration. All pathological evaluations were performed by a single experienced pathologist who was blinded to the experimental group assignments. Each sciatic nerve segment was examined under a light microscope (Olympus BX53, Olympus Co., Tokyo, Japan), and digital imaging software (Olympus DP-22 Microscope Digital Camera Software Program, Tokyo, Japan) was used to support standardized microscopic evaluation and documentation. No automated image analysis software was used for histopathological scoring.

Myelin damage and axonal alterations were assessed under light microscopy at ×200 magnification. Axonal degeneration was identified by swelling (pale staining), shrinkage (dark staining), and vacuolar formation, while myelin abnormalities were defined by thinning, collapse, or structural disruption. Histopathological (HP) scoring was performed semi-quantitatively using a previously validated grading scale ranging from 0 to 3 (0 = normal, 1 = mild, 2 = moderate, 3 = severe) [20].

The number and morphological features of axons in the sciatic nerve sections were also evaluated. For each animal, transverse sections obtained at a standardized level of the harvested sciatic nerve segment were assessed. Five non-overlapping microscopic fields were randomly selected from each section and examined at ×100 magnification using the Olympus DP-22 Microscope Digital Camera Software Program. Axons were counted using identical predefined counting criteria and field areas for all specimens by the pathologist, who was blinded to group allocation. The mean axon count obtained from the five fields was used as the representative value for each animal. To assess intraobserver reproducibility, a randomly selected subset of sections was re-evaluated by the same pathologist under blinded conditions, and agreement was assessed using the intraclass correlation coefficient (ICC) with 95% confidence intervals. Axon counting was performed according to the previously described method [21].

Immunohistochemistry of Caspase-3 and nuclear factor kappa B

All immunohistochemical staining procedures were conducted at ambient temperature utilizing the DAKO Autostainer Universal Staining System (Autostainer Link 48 DAKO, Glostrup, Denmark). Paraffin-embedded tissue sections, precisely cut to a thickness of 4 μm, were adhered to positively charged glass slides. The sections underwent deparaffinization in xylene followed by dehydration through a graded series of ethanol solutions. Antigen retrieval was subsequently carried out in a thermostatic bath (PT Link) for a duration of 40 minutes at 96°C in 10 mM/L citrate buffer, pH 6. Following this, the sections were incubated at room temperature for 60 minutes with primary antibodies: Caspase-3 (NCL-CPP32, Clone JHM62, Vision Biosystems, Novocastra, at a 1/50 dilution) and Nuclear Factor kappa B (NF-κB, sc-7386, Santa Cruz Biotechnology, USA, at a 1/500 dilution). Positive and negative controls were incorporated into each antibody staining group to ensure accuracy. Immunoreactivity was visualized utilizing the streptavidin-biotin enhanced immunoperoxidase method (K8000 Envision Flex, DAKO, Glostrup, Denmark) in the automated staining system. The sections were then incubated with DAB, followed by light counterstaining with hematoxylin to highlight antigen-antibody binding. Lastly, the sections were dehydrated and mounted with a water-soluble mounting medium. Samples with confirmed light positive immunoreactivity served as positive controls, while normal rabbit serum IgG was used as a negative control, substituting the primary antibody.

To quantify the proportion of Caspase-3 positive cells, the scoring methodology formulated by Schmeichel et al. was employed [22]. The method ranged from 0 to 4, with the following categories: <5%, 6-15%, 16-25%, 26-35%, and >35%.

To assess the proportion of NF-κB positive cells, a semi-quantitative grading system was implemented to evaluate the immunostaining of both axonal structures and Schwann cells labeled by the NF-κB antibody (Grade 0: <2%, Grade 1: 2-15%, Grade 2: 16-25%, Grade 3: 26-35%, Grade 4: ≥36%) [23].

Statistical analysis

Statistical analysis of the data was conducted using IBM SPSS Statistics for Windows, Version 22 (Released 2013; IBM Corp., Armonk, New York, United States). Data distribution was assessed using the Kolmogorov-Smirnov test. SFI values measured on days 15 and 30 were compared among the Control (C), Control + Pirfenidone (CP), Injury (I), and Injury + Pirfenidone (IP) groups separately at each time point using the Kruskal-Wallis test. When the overall test was significant, pairwise comparisons were performed using the Mann-Whitney U test with Bonferroni correction for the six possible pairwise comparisons among the four groups. Because SFI measurements on days 15 and 30 were obtained from the same animals, within-group changes between the two time points were assessed using the Wilcoxon signed-rank test. Axon numbers were compared among the four groups using one-way analysis of variance (ANOVA), followed by Tamhane’s T2 post hoc test when appropriate. Histopathological grading, Caspase-3 scores, and NF-κB scores were compared between the I and IP groups using the Mann-Whitney U test. For the principal I versus IP comparisons, effect sizes were reported with 95% confidence intervals. Cohen’s d was used for SFI and axon-count comparisons, whereas rank-biserial correlation was used for histopathological grading, Caspase-3, and NF-κB comparisons. Intraobserver reproducibility of axon counting was assessed using the ICC with 95% confidence intervals. Descriptive statistics were expressed as mean ± standard deviation for normally distributed variables and as median (25th-75th percentiles) for non-normally distributed variables. All statistical tests were two-sided, and a p-value <0.05 was considered statistically significant, with Bonferroni adjustment applied where specified for multiple pairwise comparisons.

## Results

Walking track analysis results

The comparison of SFI values among the C, CP, I, and IP groups on days 15 and 30 is presented in Table 1. Overall differences among the four groups were statistically significant at both time points (Kruskal-Wallis test, p<0.001 for both).

**Table 1:** Sciatic functional index values according to the study group and time point Data are presented as mean ± standard deviation. SFI, Sciatic Functional Index; C, Control; CP, Control + Pirfenidone; I, Injury; IP, Injury + Pirfenidone; CI, confidence interval. *Overall between-group comparisons at each time point were performed using the Kruskal-Wallis test. Pairwise I vs. IP comparisons were performed using the Mann-Whitney U test with Bonferroni correction for the six possible pairwise comparisons. † Effect sizes for the I vs. IP comparisons are reported as Cohen’s d with 95% confidence intervals. Positive values indicate higher SFI values in the IP group. ‡ Within-group comparisons between days 15 and 30 were performed using the Wilcoxon signed-rank test.

SFI	C (n=8)	CP (n=8)	I (n=8)	IP (n=8)	Overall p*	I vs. IP p	Cohen’s d (95% CI)†
Day 15	−8.6 ± 1.1	−8.7 ± 1.0	−82.11 ± 9.56	−75.16 ± 4.64	<0.001	0.516	0.87 (−0.17 to 1.88)
Day 30	−8.9 ± 1.3	−8.5 ± 1.2	−49.25 ± 7.78	−25.53 ± 4.16	<0.001	<0.001	3.59 (1.82 to 5.31)
Within-group p‡	0.641	0.742	0.008	0.008	—	—	—

On day 15, both injured groups (I and IP) had significantly lower SFI values than the non-injured control groups (C and CP) after correction for multiple comparisons (all adjusted p<0.001). No significant difference was observed between the C and CP groups (adjusted p=1.000). Although the IP group showed a numerically higher SFI value than the I group, the difference was not statistically significant after correction for multiple comparisons (adjusted p=0.516; Cohen’s d=0.87, 95% CI: −0.17 to 1.88).

On day 30, no significant difference was observed between the C and CP groups (adjusted p=1.000), and both injured groups continued to have significantly lower SFI values than the non-injured groups (all adjusted p<0.001). Importantly, the IP group had significantly higher SFI values than the I group (adjusted p<0.001), with a large standardized between-group difference (Cohen’s d=3.59, 95% CI: 1.82-5.31).

Because SFI measurements on days 15 and 30 were obtained from the same animals, within-group longitudinal changes were also evaluated using the Wilcoxon signed-rank test. No significant change in the SFI was observed between days 15 and 30 in the C (p=0.641) or CP (p=0.742) groups. In contrast, SFI values improved significantly from day 15 to day 30 in both the I and IP groups (p=0.008 for both).

Histopathological results

For the histopathological evaluation of rat sciatic nerves, hematoxylin-eosin staining was performed to assess general morphology and structural alterations. In transverse sections, axons were centrally located and surrounded by Schwann cells and endoneurial tissue.

In the control groups (C and CP), the axonal architecture and myelin sheaths appeared regular, with no evidence of axonal shrinkage, swelling, or structural disruption.

In contrast, the Injury (I) group demonstrated marked histopathological alterations, including prominent axonal degeneration, vacuolization, and disruption of myelin integrity. The lamellar structure of the myelin sheath was disorganized, and some axons appeared reduced in size or completely degenerated. In areas of severe damage, nerve fiber disintegration and inflammatory cell infiltration, including macrophage activity, were observed.

The Injury + Pirfenidone (IP) group exhibited similar pathological features; however, the severity of axonal degeneration, vacuolization, and myelin disruption was notably reduced compared to the I group. These findings suggest a partial protective effect of pirfenidone on nerve tissue following injury.

The representative histopathological features of all groups are shown in Figure 4.

**Figure 4:**
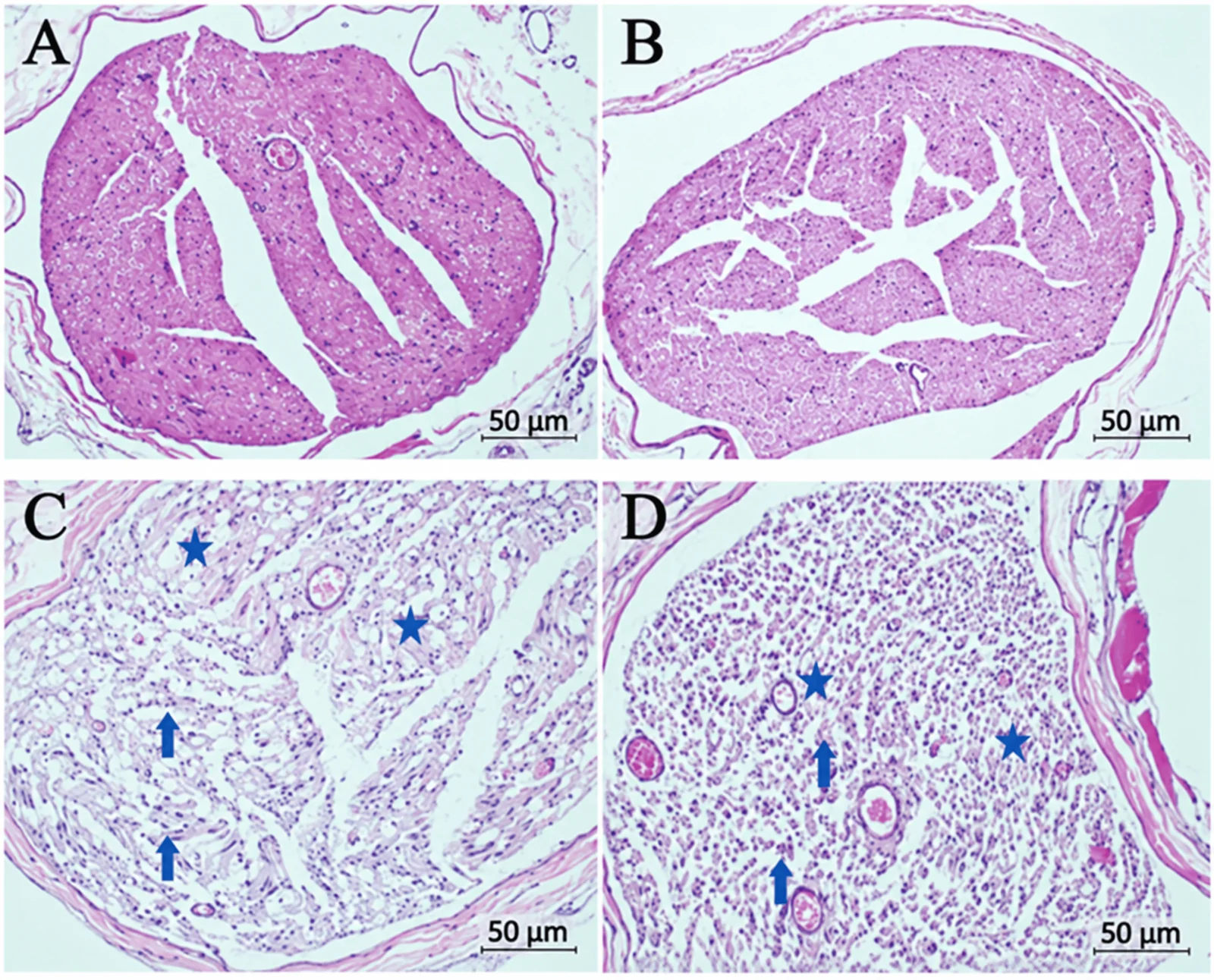
Representative histopathological images of rat sciatic nerves (hematoxylin-eosin staining, ×200). (A) Control (C) group, demonstrating normal nerve architecture; (B) Control + Pirfenidone (CP) group, demonstrating normal nerve architecture; (C) Injury (I) group, showing prominent axonal degeneration, vacuolization (asterisks), and myelin disruption (arrows); (D) Injury + Pirfenidone (IP) group, exhibiting reduced structural damage compared with the Injury group, with partial preservation of nerve architecture

Number of axons

The comparison of axon counts among the groups is presented in Table 2. Axon counts were significantly higher in the C and CP groups than in the I and IP groups (p<0.001). Although the mean axon count was higher in the IP group than in the I group, the difference did not reach statistical significance (p>0.05; Cohen’s d=1.20, 95% CI: −0.01 to 2.34). Intraobserver reproducibility of axon counting was excellent (ICC=0.94, 95% CI: 0.88-0.97).

**Table 2:** Comparison of axon counts among the study groups Data are presented as mean ± standard deviation. C, Control; CP, Control + Pirfenidone; I, Injury; IP, Injury + Pirfenidone; CI, confidence interval. * Overall between-group comparison was performed using one-way analysis of variance (ANOVA), followed by Tamhane’s T2 post hoc test. † Effect size for the I vs. IP comparison is reported as Cohen’s d with a 95% confidence interval. A positive value indicates a higher axon count in the IP group.

Parameter	C (n=8)	CP (n=8)	I (n=8)	IP (n=8)	Overall p*	I vs. IP p	Cohen’s d (95% CI)†
Axon count	239.0 ± 1.85	239.3 ± 2.38	174.5 ± 12.17	190.9 ± 13.68	<0.001	>0.05	1.20 (−0.01 to 2.34)

Caspase-3 immunostaining

Caspase-3 immunostaining was detected in both axonal structures and Schwann cells. The stained sections were meticulously analyzed under a magnification of ×400. Caspase-3 expression was absent in both the C and CP groups (Figure 5A and Figure 5B, respectively). In contrast, Caspase-3 immunoreactivity was notably elevated in the I group (Figure 5C), while a discernible but comparatively weaker staining intensity was observed in the IP group (Figure 5D).

**Figure 5:**
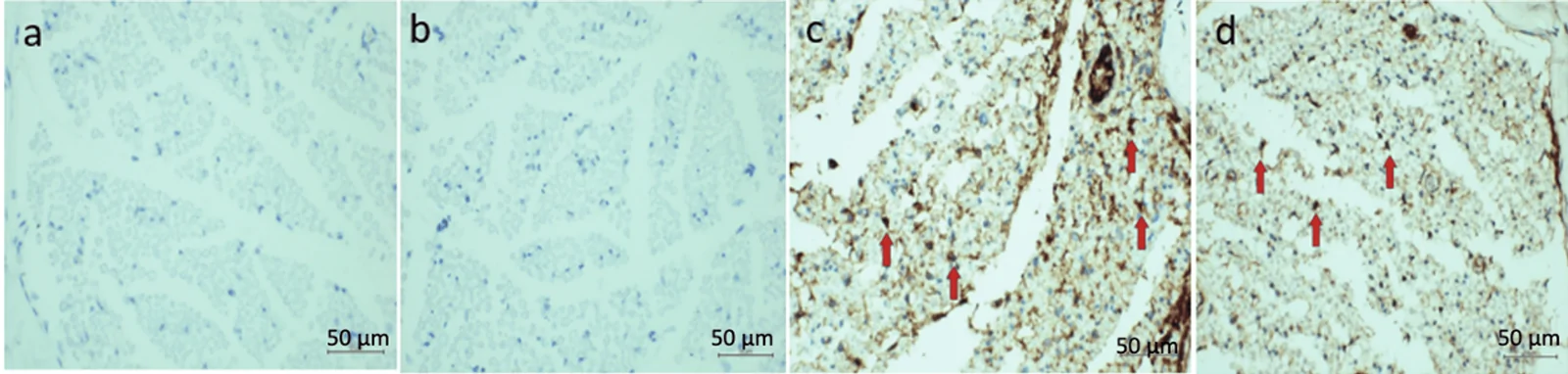
Representative images of Caspase-3 immunohistochemical staining (× 400) in groups C (a), CP (b), I (c) and IP (d). Immunostaining in axons and Schwann cells (arrows) C, Control; CP, Control + Pirfenidone; I, Injury; IP, Injury + Pirfenidone

NF-κB immunostaining 

NF-κB immunostaining was identified in both axonal structures and Schwann cells. No discernible staining was observed in the C and CP groups (Figure 6A and Figure 6B, respectively). NF-κB immunoreactivity was observed in the I group (Figure 6C) and was descriptively lower in the IP group (Figure 6D). However, the difference in NF-κB scores between the I and IP groups was not statistically significant (p=0.161; rank-biserial correlation=−0.40, 95% CI: −0.75 to 0.13), as shown in Table 3.

**Figure 6:**
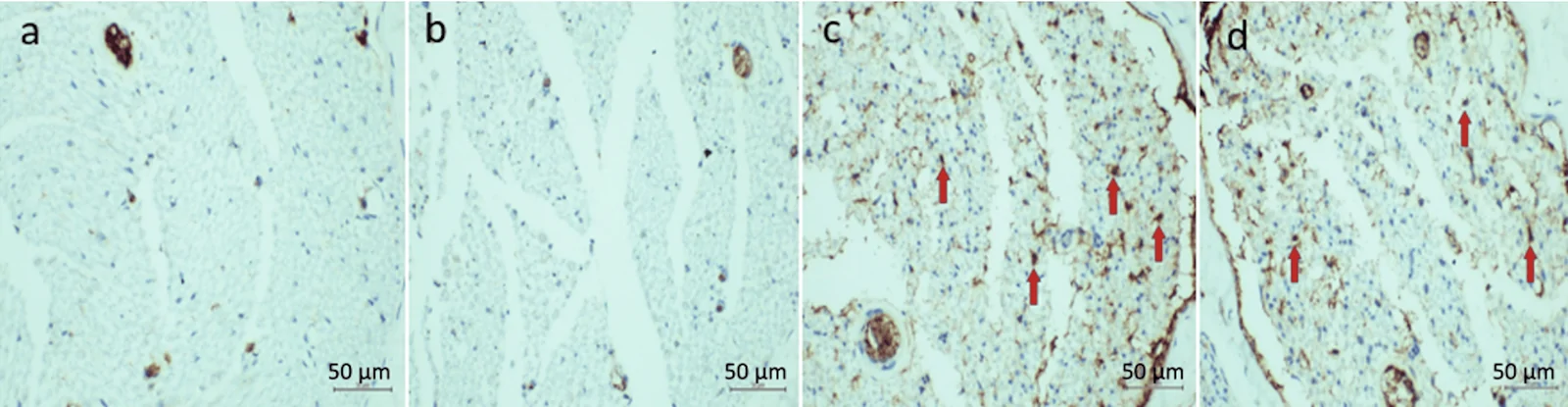
Representative images of NF-kB immunohistochemical staining (× 400) in groups C (a), CP (b), I (c) and IP (d). Immunostaining in axons and Schwann cells (arrows) C, Control; CP, Control + Pirfenidone; I, Injury; IP, Injury + Pirfenidone

**Table 3:** Comparison of histopathological and immunohistochemical findings between the Injury and Injury + Pirfenidone groups Data are presented as median (25th–75th percentiles). I, Injury; IP, Injury + Pirfenidone; NF-κB, nuclear factor kappa B; CI, confidence interval. * Comparisons between the I and IP groups were performed using the Mann-Whitney U test. † Effect sizes are reported as rank-biserial correlations with 95% confidence intervals. Negative values indicate lower histopathological or immunohistochemical scores in the IP group relative to the I group.

Parameter	I (n=8)	IP (n=8)	p*	Rank-biserial correlation (95% CI)†
Histopathological score	2.50 (2.00–3.00)	2.00 (2.00–2.75)	0.442	−0.25 (−0.67 to 0.29)
Caspase-3 score	3.00 (2.00–3.00)	2.00 (1.25–3.00)	0.195	−0.35 (−0.72 to 0.18)
NF-κB score	2.00 (2.00–3.00)	1.00 (1.00–2.00)	0.161	−0.40 (−0.75 to 0.13)

## Discussion

In the present study, pirfenidone administration was associated with improved functional recovery following experimental sciatic nerve injury, as demonstrated by significantly higher SFI values in the Injury + Pirfenidone group than in the Injury group on day 30. In contrast, no statistically significant differences were observed between these groups in axon count, histopathological injury grading, Caspase-3 immunoreactivity, or NF-κB immunoreactivity. Therefore, although some histopathological and immunohistochemical findings were numerically more favorable in the pirfenidone-treated group, these findings do not establish a tissue-protective, anti-inflammatory, anti-apoptotic, or antifibrotic mechanism.

Pirfenidone has been approved for the treatment of idiopathic pulmonary fibrosis and is known to have pleiotropic antifibrotic, anti-inflammatory, and antioxidative properties [24-27]. For this reason, it has increasingly been investigated in different experimental models of tissue injury [25,28-31]. However, evidence regarding its effects on peripheral nerve injury remains limited. Peripheral nerve healing is a multifactorial process involving inflammatory responses, axonal degeneration, Schwann cell-related changes, extracellular matrix remodeling, and scar formation. Accordingly, pirfenidone was investigated in the present study because of its broad pharmacological profile. Although Masson’s trichrome staining suggested increased collagen fibers in the Injury group, a dedicated quantitative assessment of fibrosis was not performed. Therefore, the present findings cannot determine whether an antifibrotic effect contributed to the observed functional improvement.

In 2024, Abo-Elsoud et al. [32] demonstrated that pirfenidone mitigated several alterations associated with cuprizone-induced demyelination in rats. Pirfenidone administration increased sciatic nerve conduction velocity and the distance traveled in the open-field test. Abo-Elsoud et al. [32] also reported increased superoxide dismutase, nerve growth factor, and neuregulin-1 levels and reduced malondialdehyde, TNF-α, transforming growth factor beta (TGF-β), interferon gamma (IFN-γ), and NF-κB levels. These findings provide a biological rationale for investigating pirfenidone in neural injury models. However, the cuprizone model differs substantially from the traumatic sciatic nerve injury model used in the present study, and these previously reported mechanisms cannot be directly extrapolated to our findings.

Caspase-3 and NF-κB were evaluated in the present study as exploratory markers of apoptotic and inflammatory responses, respectively. Although both scores were numerically lower in the Injury + Pirfenidone group, neither difference reached statistical significance. The Caspase-3 score was lower in the pirfenidone-treated group, but the between-group difference was not statistically significant (p=0.195; rank-biserial correlation=−0.35, 95% CI: −0.72 to 0.18). Similarly, NF-κB immunoreactivity was numerically lower in the Injury + Pirfenidone group without reaching statistical significance (p=0.161; rank-biserial correlation=−0.40, 95% CI: −0.75 to 0.13). Accordingly, the present data do not demonstrate anti-apoptotic or anti-inflammatory effects of pirfenidone. Previous experimental studies have nevertheless reported modulation of these pathways by pirfenidone in other tissue-injury models [16,33]. Mersal et al. reported reductions in TNF-α and IL-1β levels and anti-Caspase-3 activity with pirfenidone in a rat model of methotrexate-induced testiculopathy [34], while pirfenidone has also been reported to prevent corneal fibrosis in association with increased nerve growth factor expression [35]. These studies identify potential biological pathways for future investigation but should not be interpreted as evidence that the functional improvement observed in our study occurred through the same mechanisms.

NF-κB remains relevant to peripheral nerve injury because it is involved in inflammatory signaling and cellular responses after nerve damage [36-40]. Wang et al. demonstrated an association between NF-κB expression and apoptotic processes in Schwann cells following sciatic nerve injury in rats [36]. Other experimental studies have investigated modulation of NF-κB signaling as a potential approach to peripheral nerve recovery [37-40]. For example, Zhang et al. reported that paeoniflorin inhibited NF-κB signaling and reduced Schwann cell apoptosis in an experimental rat model [38]. Sun et al. reported that TNF-α-induced protein 8-like 2 was associated with NF-κB inactivation, changes in inflammatory cytokines, and improved outcomes following sciatic nerve injury [40]. These studies support the relevance of NF-κB as a candidate pathway in peripheral nerve injury but do not establish NF-κB modulation as the mechanism responsible for the SFI improvement observed with pirfenidone in the present study.

Previous studies have also associated suppression of NF-κB signaling with changes in pain and recovery following experimental sciatic nerve injury [41-44]. Pirfenidone has been reported to influence TNF-α-related inflammatory signaling in other experimental settings [26]. In addition, IL-10 has an important role in regulating inflammatory responses following tissue and peripheral nerve injury [45,46]. Palathingal Bava et al. reported increased IL-10 release following pirfenidone administration in experimental acute pancreatitis [47], whereas Mietto et al. demonstrated impaired resolution of inflammation and axonal regeneration in IL-10-deficient rats following peripheral nerve injury [46]. Although these observations provide possible pathways for future mechanistic studies of pirfenidone, TNF-α, IL-10, and related cytokines were not measured in the present study. Consequently, their contribution to the observed functional findings cannot be determined from our data.

A notable finding of the present study was the time-dependent difference in functional recovery. On day 15, SFI did not differ significantly between the Injury and Injury + Pirfenidone groups after correction for multiple comparisons (adjusted p=0.516; Cohen’s d=0.87, 95% CI: −0.17 to 1.88). By day 30, however, SFI was significantly higher in the Injury + Pirfenidone group (adjusted p<0.001; Cohen’s d=3.59, 95% CI: 1.82-5.31). Peripheral nerve regeneration is a time-dependent process in which early Wallerian degeneration and inflammatory responses are followed by Schwann cell-mediated repair, axonal regrowth, and remyelination [14,48-50]. The emergence of a between-group difference at day 30 may therefore reflect the temporal course of functional recovery; however, the present study was not designed to determine which biological process accounted for this difference.

Pirfenidone administration was initiated immediately after nerve injury to evaluate its effects throughout the early post-injury period and subsequent regenerative process. This timing was selected because the initial phase after peripheral nerve injury involves early inflammatory and degenerative responses that may influence later regeneration. However, the present study did not include a delayed-treatment group; therefore, it cannot determine whether treatment initiated at a later stage would produce similar functional outcomes.

Overall, the principal finding of the present study is that pirfenidone administration was associated with improved functional recovery at day 30 after experimental sciatic nerve injury. The absence of statistically significant between-group differences in histopathological grading, axon count, Caspase-3, and NF-κB prevents attribution of this functional improvement to a specific tissue-protective, anti-inflammatory, or anti-apoptotic mechanism. Furthermore, because fibrosis was not quantitatively assessed, an antifibrotic mechanism cannot be established. Future studies incorporating quantitative fibrosis assessment, inflammatory and apoptotic biomarkers, electrophysiological evaluation, and multiple treatment doses are needed to determine the biological mechanisms underlying the observed functional difference.

Limitations

This study has several limitations. First, electrophysiological assessments, such as nerve conduction studies, were not performed and could have provided complementary information regarding nerve recovery. In addition, inflammatory biomarkers such as TNF-α, IL-1β, IL-6, IFN-γ, IL-17, TGF-β, and IL-10, as well as oxidative stress markers such as glutathione, malondialdehyde, and nitrite/nitrate levels, were not evaluated. Therefore, the present study cannot determine whether anti-inflammatory or antioxidant mechanisms contributed to the observed functional improvement. RT-PCR analysis of NF-κB, neuregulin-1, and nerve growth factor expression could have provided additional molecular information. Furthermore, neurotrophic and regeneration-associated signaling pathways such as BDNF, ERK, and AKT were not evaluated, limiting mechanistic interpretation of the observed findings.

Another important limitation is the absence of quantitative fibrosis assessment. Although Masson’s trichrome staining provided morphological information regarding collagen deposition in the injured nerves, collagen area fraction, fibroblast/myofibroblast activation, and extracellular matrix remodeling markers such as collagen I/III, fibronectin, α-SMA, MMPs, and TIMPs were not quantitatively evaluated. Therefore, the present study cannot determine the contribution of fibrosis to impaired nerve regeneration or establish an antifibrotic mechanism for pirfenidone.

In addition, pirfenidone was not compared with another active antifibrotic or neuroregenerative agent, limiting conclusions regarding its relative efficacy. Only a single dose of pirfenidone was evaluated; therefore, a dose-response relationship could not be determined, and the optimal dose for peripheral nerve injury remains unknown. Specific cell populations involved in nerve repair, including Schwann cells, fibroblasts, macrophages, and endothelial cells, were also not separately evaluated. Consequently, cell type-specific effects of pirfenidone could not be determined.

## Conclusions

In this experimental sciatic nerve injury model, pirfenidone administration was associated with significantly improved functional recovery at day 30. Although Caspase-3 and NF-κB immunoreactivity was numerically lower in the pirfenidone-treated group, these differences were not statistically significant and therefore do not establish anti-apoptotic or anti-inflammatory effects. Furthermore, because fibrosis was not quantitatively assessed, an antifibrotic mechanism cannot be established from the present findings. Further studies incorporating molecular, electrophysiological, and quantitative fibrosis assessments are needed to clarify the mechanisms underlying the observed functional improvement.
